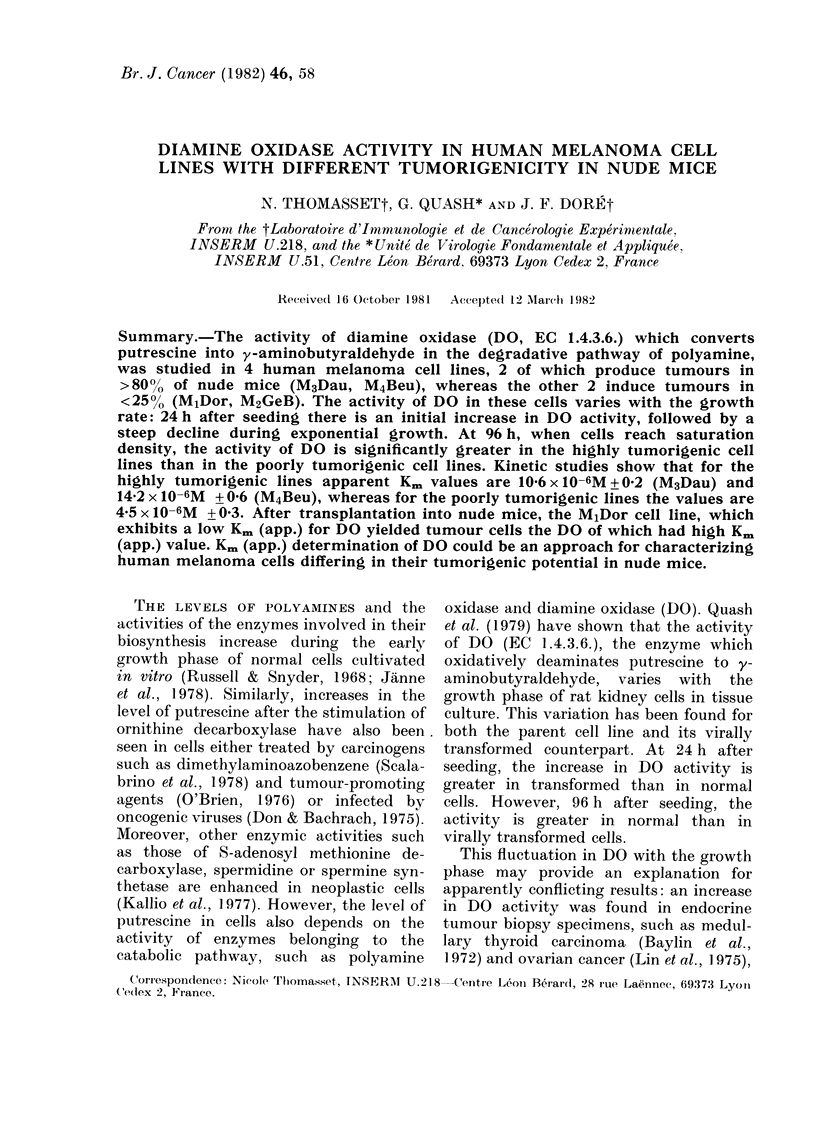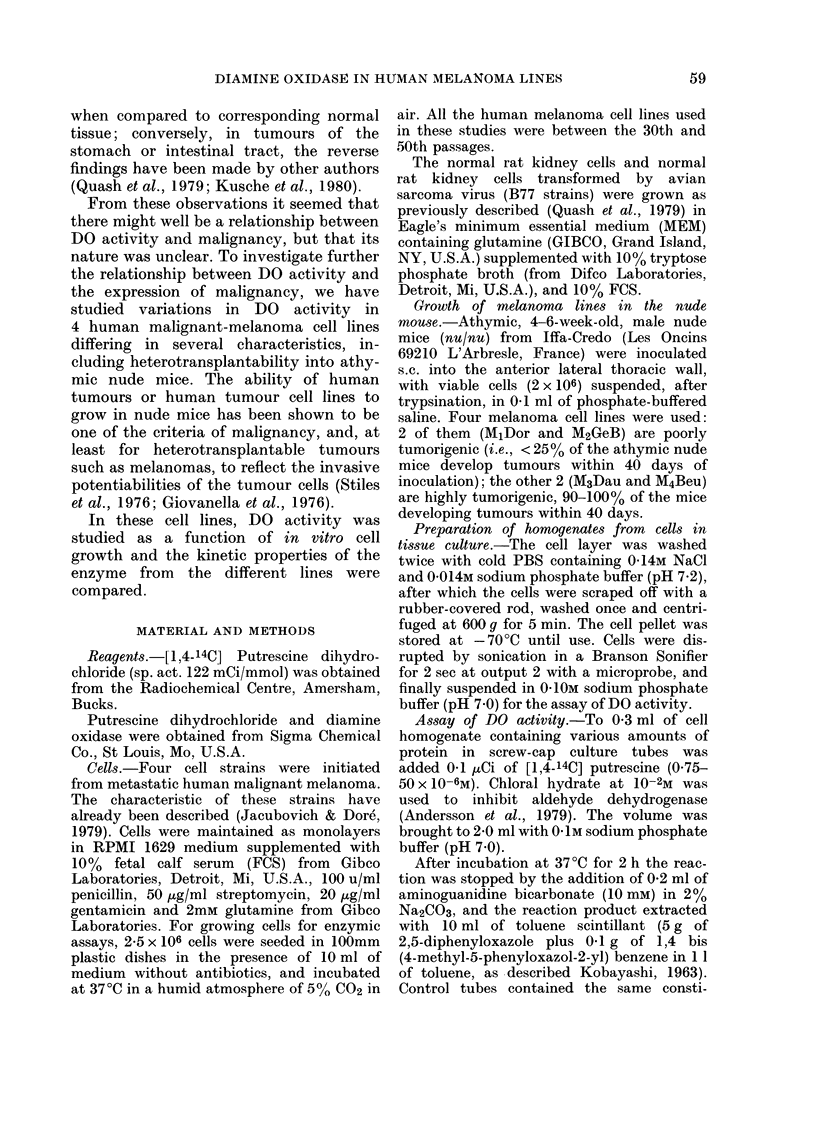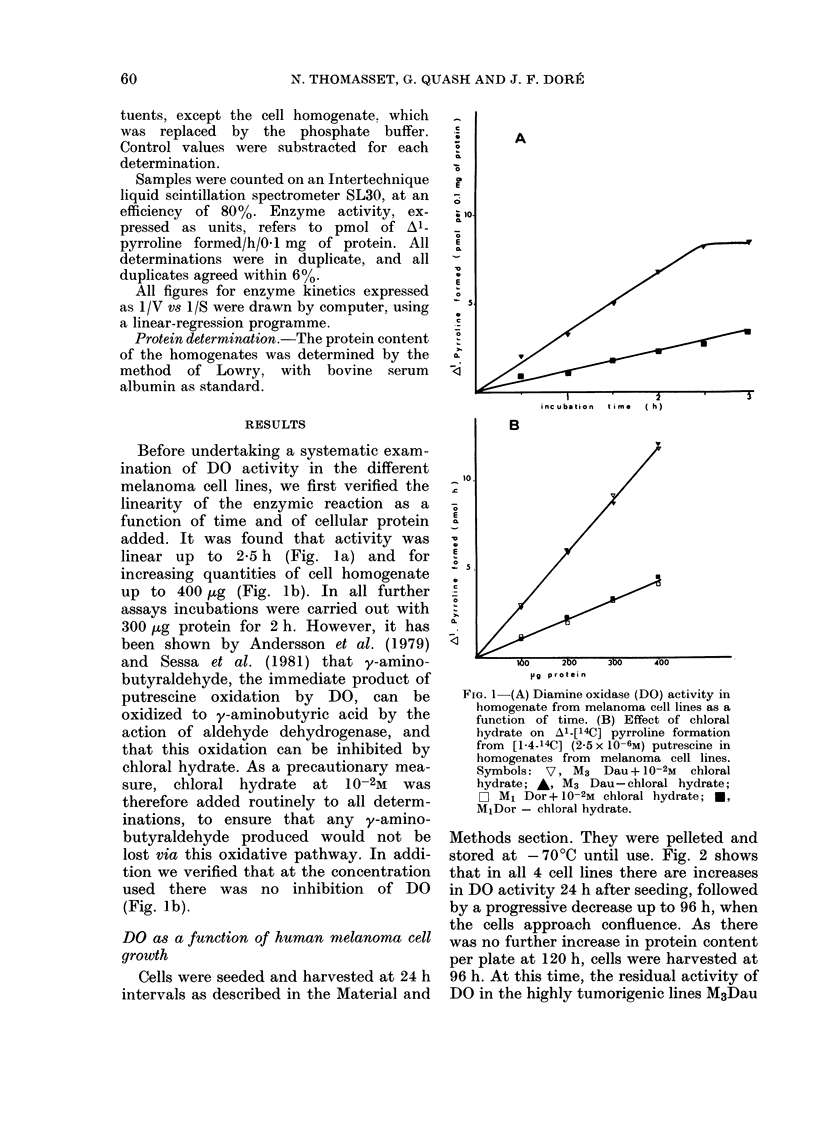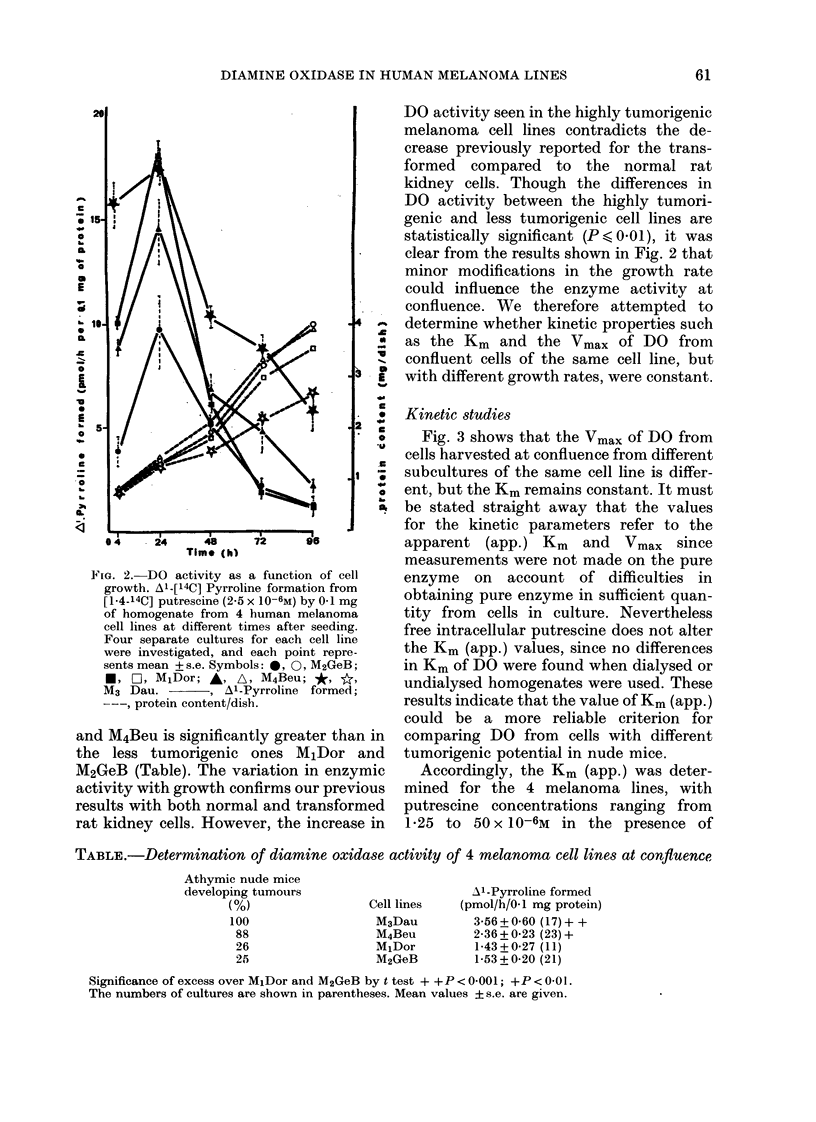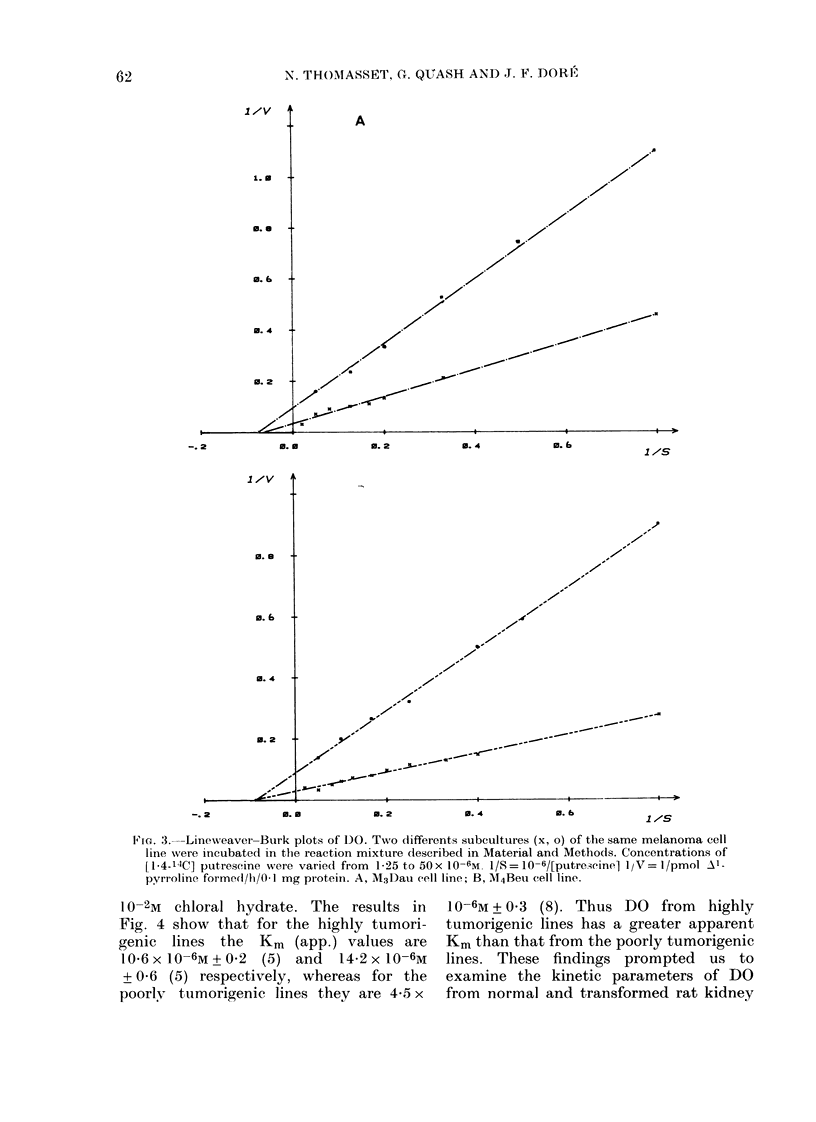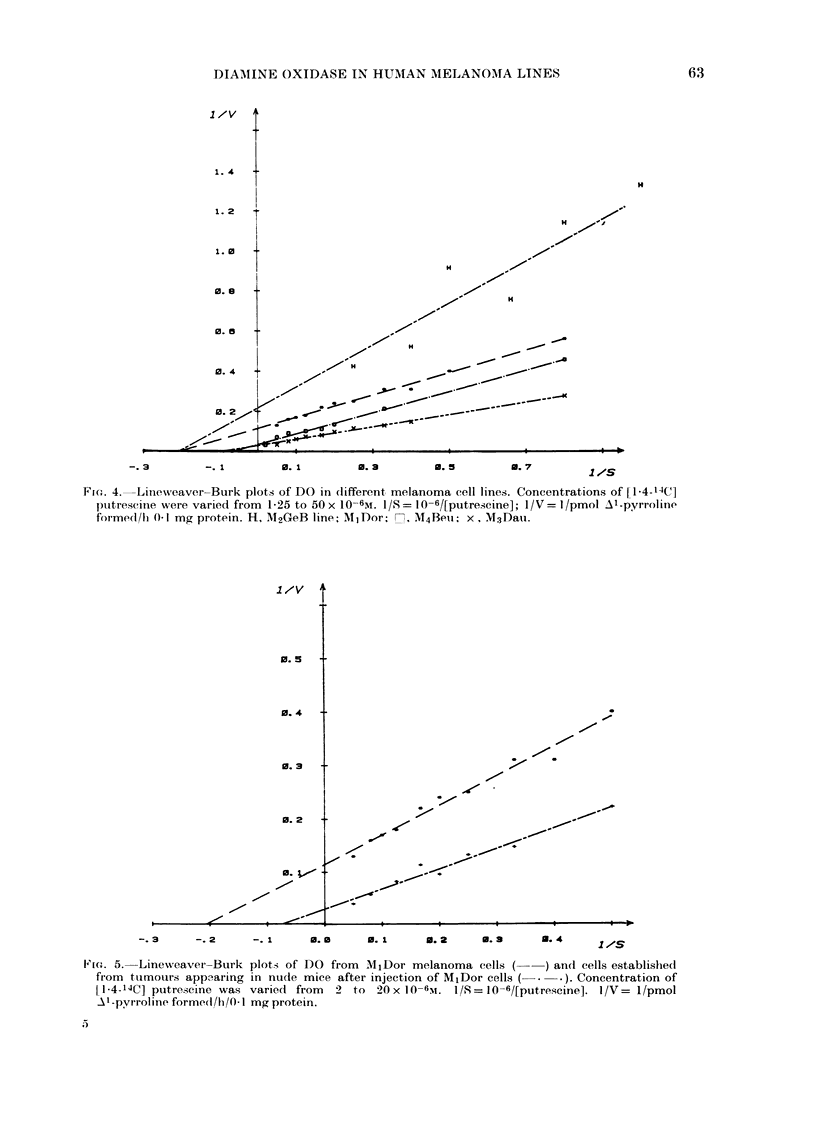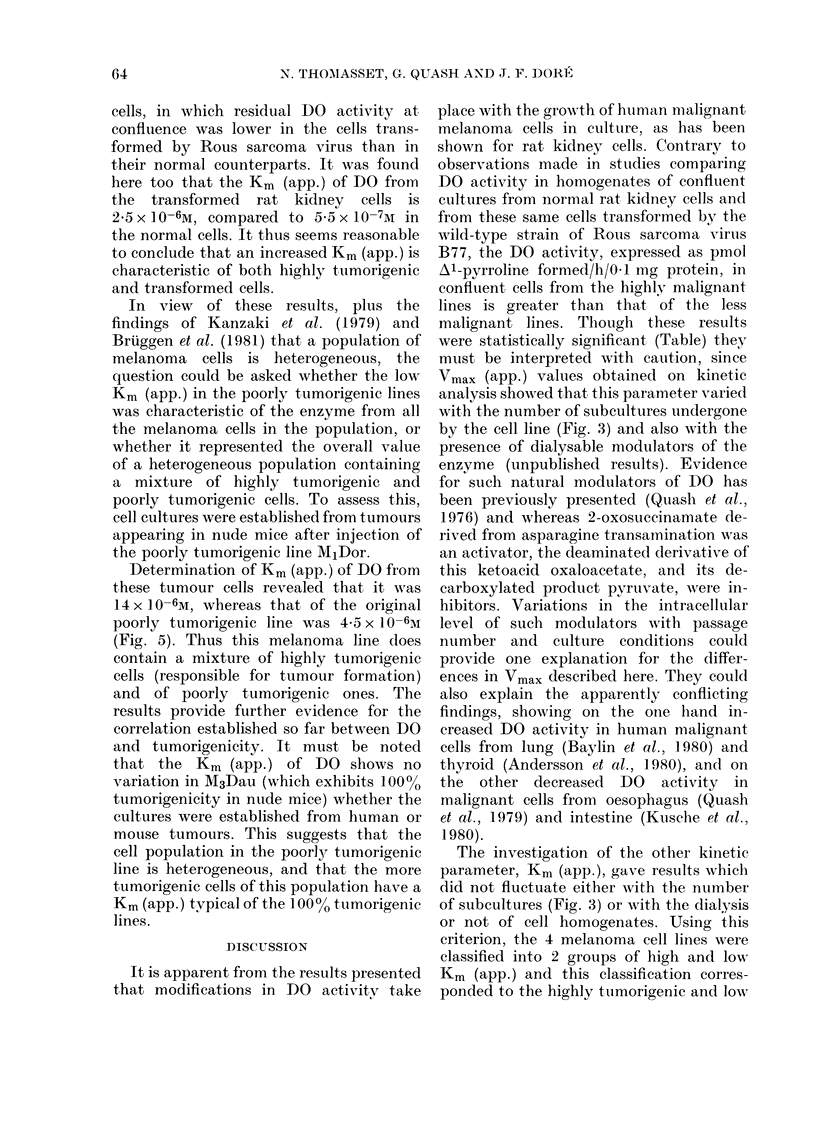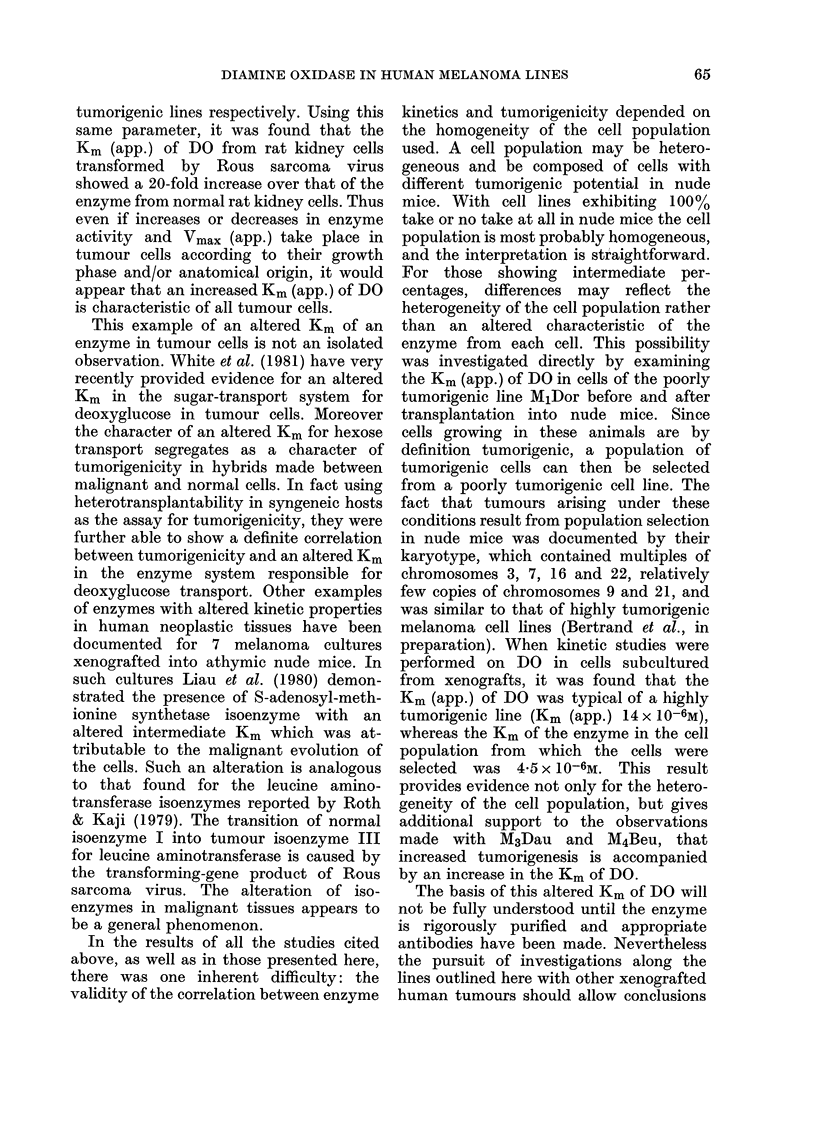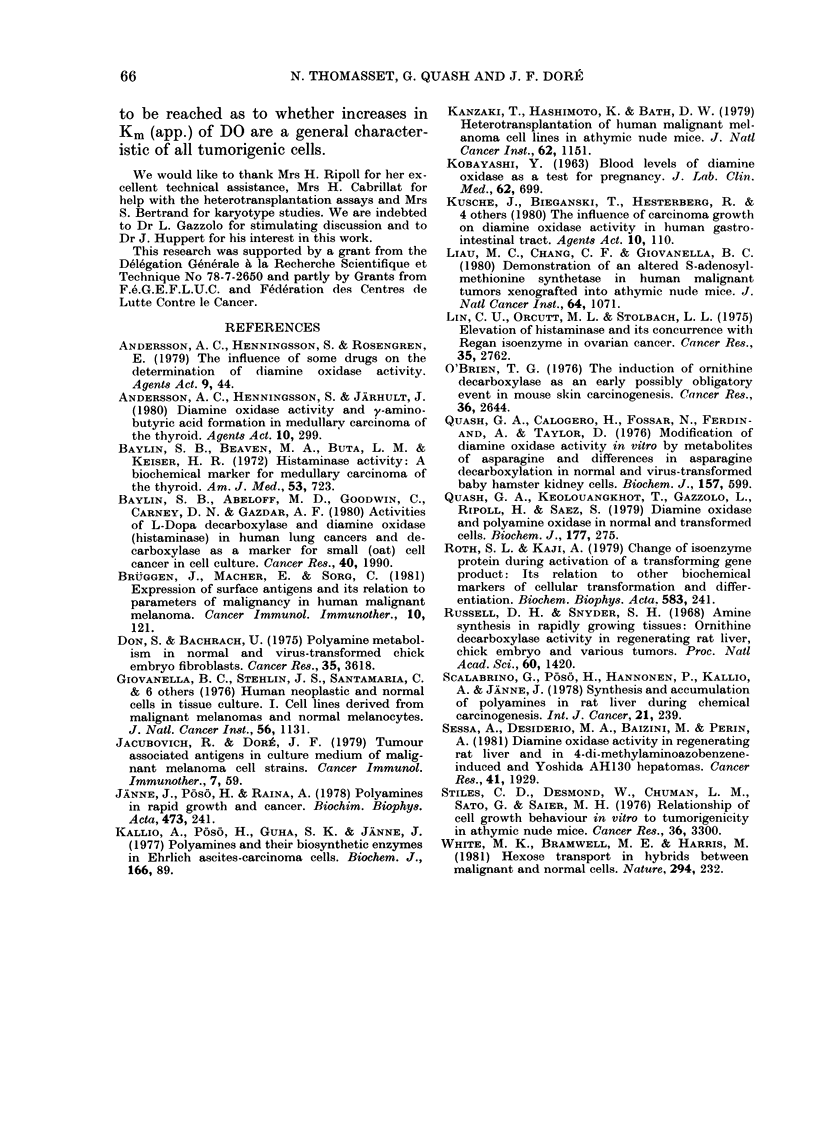# Diamine oxidase activity in human melanoma cell lines with different tumorigenicity in nude mice.

**DOI:** 10.1038/bjc.1982.165

**Published:** 1982-07

**Authors:** N. Thomasset, G. Quash, J. F. Doré

## Abstract

The activity of diamine oxidase (DO, EC 1.4.3.6.) which converts putrescine into gamma-aminobutyraldehyde in the degradative pathway of polyamine, was studied in 4 human melanoma cell lines, 2 of which produce tumours in greater than 80% of nude mice (M3Dau, M4Beu), whereas the other 2 induce tumours in less than 25% (M1Dor, M2GeB). The activity of DO in these cells varies with the growth rate: 24 h after seeding there is an initial increase in DO activity, followed by a steep decline during exponential growth. At 96 h, when cells reach saturation density, the activity of DO is significantly greater in the highly tumorigenic cell lines than in the poorly tumorigenic cell lines. Kinetic studies show that for the highly tumorigenic lines apparent Km values are 10.6 X 10(-6)M +/- 0.2 (M3Dau) and 14.2 X 10(-6) M +/- 0.6 (M4Beu), whereas for the poorly tumorigenic lines the values are 4.5 X 10(-6) M +/- 0.3. After transplantation into nude mice, the M1Dor cell line, which exhibits a low Km (app.) for DO of which had high Km (app.) value. Km (app.) determination of DO could be an approach for characterizing human melanoma cells differing in their tumorigenic potential in nude mice.


*Br. J. Cancer* (1982) **46**, 58

### DIAMINE OXIDASE ACTIVITY IN HUMAN MELANOMA CELL LINES WITH DIFFERENT TUMORIGENICITY IN NUDE MICE

N. THOMASSET†, G. QUASH* AND J. F. DORÉ†

*From the †Laboratoire d'Immunologie et de Cancérologie Expérimentale, INSERM U.218, and the *Unité de Virologie Fondamentale et Appliquée, INSERM U.51, Centre Léon Bérard, 69373 Lyon Cedex 2, France*

Received 16 October 1981 Accepted 12 March 1982

**Summary.—The activity of diamine oxidase (DO, EC 1.4.3.6.) which converts putrescine into γ-aminobutyraldehyde in the degradative pathway of polyamine, was studied in 4 human melanoma cell lines, 2 of which produce tumours in >80% of nude mice ($M_3$Dau, $M_4$Beu), whereas the other 2 induce tumours in <25% ($M_1$Dor, $M_2$GeB). The activity of DO in these cells varies with the growth rate: 24 h after seeding there is an initial increase in DO activity, followed by a steep decline during exponential growth. At 96 h, when cells reach saturation density, the activity of DO is significantly greater in the highly tumorigenic cell lines than in the poorly tumorigenic cell lines. Kinetic studies show that for the highly tumorigenic lines apparent $K_m$ values are $10{\cdot}6 \times 10^{-6}$M $\pm 0{\cdot}2$ ($M_3$Dau) and $14{\cdot}2 \times 10^{-6}$M $\pm 0{\cdot}6$ ($M_4$Beu), whereas for the poorly tumorigenic lines the values are $4{\cdot}5 \times 10^{-6}$M $\pm 0{\cdot}3$. After transplantation into nude mice, the $M_1$Dor cell line, which exhibits a low $K_m$ (app.) for DO yielded tumour cells the DO of which had high $K_m$ (app.) value. $K_m$ (app.) determination of DO could be an approach for characterizing human melanoma cells differing in their tumorigenic potential in nude mice.**

THE LEVELS OF POLYAMINES and the activities of the enzymes involved in their biosynthesis increase during the early growth phase of normal cells cultivated *in vitro* (Russell & Snyder, 1968; Jänne *et al.*, 1978). Similarly, increases in the level of putrescine after the stimulation of ornithine decarboxylase have also been seen in cells either treated by carcinogens such as dimethylaminoazobenzene (Scalabrino *et al.*, 1978) and tumour-promoting agents (O'Brien, 1976) or infected by oncogenic viruses (Don & Bachrach, 1975). Moreover, other enzymic activities such as those of S-adenosyl methionine decarboxylase, spermidine or spermine synthetase are enhanced in neoplastic cells (Kallio *et al.*, 1977). However, the level of putrescine in cells also depends on the activity of enzymes belonging to the catabolic pathway, such as polyamine oxidase and diamine oxidase (DO). Quash *et al.* (1979) have shown that the activity of DO (EC 1.4.3.6.), the enzyme which oxidatively deaminates putrescine to γ-aminobutyraldehyde, varies with the growth phase of rat kidney cells in tissue culture. This variation has been found for both the parent cell line and its virally transformed counterpart. At 24 h after seeding, the increase in DO activity is greater in transformed than in normal cells. However, 96 h after seeding, the activity is greater in normal than in virally transformed cells.

This fluctuation in DO with the growth phase may provide an explanation for apparently conflicting results: an increase in DO activity was found in endocrine tumour biopsy specimens, such as medullary thyroid carcinoma (Baylin *et al.*, 1972) and ovarian cancer (Lin *et al.*, 1975),

Correspondence: Nicole Thomasset, INSERM U.218—Centre Léon Bérard, 28 rue Laënnec, 69373 Lyon Cedex 2, France.

when compared to corresponding normal tissue; conversely, in tumours of the stomach or intestinal tract, the reverse findings have been made by other authors (Quash *et al.*, 1979; Kusche *et al.*, 1980).

From these observations it seemed that there might well be a relationship between DO activity and malignancy, but that its nature was unclear. To investigate further the relationship between DO activity and the expression of malignancy, we have studied variations in DO activity in 4 human malignant-melanoma cell lines differing in several characteristics, including heterotransplantability into athymic nude mice. The ability of human tumours or human tumour cell lines to grow in nude mice has been shown to be one of the criteria of malignancy, and, at least for heterotransplantable tumours such as melanomas, to reflect the invasive potentiabilities of the tumour cells (Stiles *et al.*, 1976; Giovanella *et al.*, 1976).

In these cell lines, DO activity was studied as a function of *in vitro* cell growth and the kinetic properties of the enzyme from the different lines were compared.

### MATERIAL AND METHODS

*Reagents.*—[1,4-$^{14}$C] Putrescine dihydrochloride (sp. act. 122 mCi/mmol) was obtained from the Radiochemical Centre, Amersham, Bucks.

Putrescine dihydrochloride and diamine oxidase were obtained from Sigma Chemical Co., St Louis, Mo, U.S.A.

*Cells.*—Four cell strains were initiated from metastatic human malignant melanoma. The characteristic of these strains have already been described (Jacubovich & Doré, 1979). Cells were maintained as monolayers in RPMI 1629 medium supplemented with 10% fetal calf serum (FCS) from Gibco Laboratories, Detroit, Mi, U.S.A., 100 u/ml penicillin, 50 $\mu$g/ml streptomycin, 20 $\mu$g/ml gentamicin and 2mM glutamine from Gibco Laboratories. For growing cells for enzymic assays, $2 \cdot 5 \times 10^6$ cells were seeded in 100mm plastic dishes in the presence of 10 ml of medium without antibiotics, and incubated at 37°C in a humid atmosphere of 5% $CO_2$ in air. All the human melanoma cell lines used in these studies were between the 30th and 50th passages.

The normal rat kidney cells and normal rat kidney cells transformed by avian sarcoma virus (B77 strains) were grown as previously described (Quash *et al.*, 1979) in Eagle's minimum essential medium (MEM) containing glutamine (GIBCO, Grand Island, NY, U.S.A.) supplemented with 10% tryptose phosphate broth (from Difco Laboratories, Detroit, Mi, U.S.A.), and 10% FCS.

*Growth of melanoma lines in the nude mouse.*—Athymic, 4–6-week-old, male nude mice (*nu/nu*) from Iffa-Credo (Les Oncins 69210 L'Arbresle, France) were inoculated s.c. into the anterior lateral thoracic wall, with viable cells ($2 \times 10^6$) suspended, after trypsination, in 0·1 ml of phosphate-buffered saline. Four melanoma cell lines were used: 2 of them ($M_1$Dor and $M_2$GeB) are poorly tumorigenic (*i.e.*, $<25\%$ of the athymic nude mice develop tumours within 40 days of inoculation); the other 2 ($M_3$Dau and $M_4$Beu) are highly tumorigenic, 90–100% of the mice developing tumours within 40 days.

*Preparation of homogenates from cells in tissue culture.*—The cell layer was washed twice with cold PBS containing 0·14M NaCl and 0·014M sodium phosphate buffer (pH 7·2), after which the cells were scraped off with a rubber-covered rod, washed once and centrifuged at 600 *g* for 5 min. The cell pellet was stored at −70°C until use. Cells were disrupted by sonication in a Branson Sonifier for 2 sec at output 2 with a microprobe, and finally suspended in 0·10M sodium phosphate buffer (pH 7·0) for the assay of DO activity.

*Assay of DO activity.*—To 0·3 ml of cell homogenate containing various amounts of protein in screw-cap culture tubes was added 0·1 $\mu$Ci of [1,4-$^{14}$C] putrescine (0·75–$50 \times 10^{-6}$M). Chloral hydrate at $10^{-2}$M was used to inhibit aldehyde dehydrogenase (Andersson *et al.*, 1979). The volume was brought to 2·0 ml with 0·1M sodium phosphate buffer (pH 7·0).

After incubation at 37°C for 2 h the reaction was stopped by the addition of 0·2 ml of aminoguanidine bicarbonate (10 mM) in 2% $Na_2CO_3$, and the reaction product extracted with 10 ml of toluene scintillant (5 g of 2,5-diphenyloxazole plus 0·1 g of 1,4 bis (4-methyl-5-phenyloxazol-2-yl) benzene in 1 l of toluene, as described Kobayashi, 1963). Control tubes contained the same constituents, except the cell homogenate, which was replaced by the phosphate buffer. Control values were substracted for each determination.

Samples were counted on an Intertechnique liquid scintillation spectrometer SL30, at an efficiency of 80%. Enzyme activity, expressed as units, refers to pmol of $\Delta^1$-pyrroline formed/h/0·1 mg of protein. All determinations were in duplicate, and all duplicates agreed within 6%.

All figures for enzyme kinetics expressed as 1/V *vs* 1/S were drawn by computer, using a linear-regression programme.

*Protein determination.*—The protein content of the homogenates was determined by the method of Lowry, with bovine serum albumin as standard.

### RESULTS

Before undertaking a systematic examination of DO activity in the different melanoma cell lines, we first verified the linearity of the enzymic reaction as a function of time and of cellular protein added. It was found that activity was linear up to 2·5 h (Fig. 1a) and for increasing quantities of cell homogenate up to 400 μg (Fig. 1b). In all further assays incubations were carried out with 300 μg protein for 2 h. However, it has been shown by Andersson *et al.* (1979) and Sessa *et al.* (1981) that γ-aminobutyraldehyde, the immediate product of putrescine oxidation by DO, can be oxidized to γ-aminobutyric acid by the action of aldehyde dehydrogenase, and that this oxidation can be inhibited by chloral hydrate. As a precautionary measure, chloral hydrate at $10^{-2}$M was therefore added routinely to all determinations, to ensure that any γ-aminobutyraldehyde produced would not be lost *via* this oxidative pathway. In addition we verified that at the concentration used there was no inhibition of DO (Fig. 1b).

FIG. 1—(A) Diamine oxidase (DO) activity in homogenate from melanoma cell lines as a function of time. (B) Effect of chloral hydrate on $\Delta^1$-[$^{14}$C] pyrroline formation from [1·4-$^{14}$C] (2·5 × $10^{-6}$M) putrescine in homogenates from melanoma cell lines. Symbols: ▽, $M_3$ Dau + $10^{-2}$M chloral hydrate; ▲, $M_3$ Dau − chloral hydrate; □ $M_1$ Dor + $10^{-2}$M chloral hydrate; ■, $M_1$Dor − chloral hydrate.

### *DO as a function of human melanoma cell growth*

Cells were seeded and harvested at 24 h intervals as described in the Material and Methods section. They were pelleted and stored at −70°C until use. Fig. 2 shows that in all 4 cell lines there are increases in DO activity 24 h after seeding, followed by a progressive decrease up to 96 h, when the cells approach confluence. As there was no further increase in protein content per plate at 120 h, cells were harvested at 96 h. At this time, the residual activity of DO in the highly tumorigenic lines $M_3$Dau

FIG. 2.—DO activity as a function of cell growth. $\Delta^1$-[$^{14}$C] Pyrroline formation from [1·4-$^{14}$C] putrescine ($2{\cdot}5 \times 10^{-6}$M) by 0·1 mg of homogenate from 4 human melanoma cell lines at different times after seeding. Four separate cultures for each cell line were investigated, and each point represents mean ± s.e. Symbols: ●, ○, $M_2$GeB; ■, □, $M_1$Dor; ▲, △, $M_4$Beu; ★, ☆, $M_3$ Dau. ———, $\Delta^1$-Pyrroline formed; - - -, protein content/dish.

and $M_4$Beu is significantly greater than in the less tumorigenic ones $M_1$Dor and $M_2$GeB (Table). The variation in enzymic activity with growth confirms our previous results with both normal and transformed rat kidney cells. However, the increase in DO activity seen in the highly tumorigenic melanoma cell lines contradicts the decrease previously reported for the transformed compared to the normal rat kidney cells. Though the differences in DO activity between the highly tumorigenic and less tumorigenic cell lines are statistically significant ($P \leqslant 0{\cdot}01$), it was clear from the results shown in Fig. 2 that minor modifications in the growth rate could influence the enzyme activity at confluence. We therefore attempted to determine whether kinetic properties such as the $K_m$ and the $V_{max}$ of DO from confluent cells of the same cell line, but with different growth rates, were constant.

*Kinetic studies*

Fig. 3 shows that the $V_{max}$ of DO from cells harvested at confluence from different subcultures of the same cell line is different, but the $K_m$ remains constant. It must be stated straight away that the values for the kinetic parameters refer to the apparent (app.) $K_m$ and $V_{max}$ since measurements were not made on the pure enzyme on account of difficulties in obtaining pure enzyme in sufficient quantity from cells in culture. Nevertheless free intracellular putrescine does not alter the $K_m$ (app.) values, since no differences in $K_m$ of DO were found when dialysed or undialysed homogenates were used. These results indicate that the value of $K_m$ (app.) could be a more reliable criterion for comparing DO from cells with different tumorigenic potential in nude mice.

Accordingly, the $K_m$ (app.) was determined for the 4 melanoma lines, with putrescine concentrations ranging from 1·25 to $50 \times 10^{-6}$M in the presence of

TABLE.—*Determination of diamine oxidase activity of* 4 *melanoma cell lines at confluence*

| Athymic nude mice developing tumours (%) | Cell lines | $\Delta^1$-Pyrroline formed (pmol/h/0·1 mg protein) |
|---|---|---|
| 100 | $M_3$Dau | 3·56 ± 0·60 (17) + + |
| 88 | $M_4$Beu | 2·36 ± 0·23 (23) + |
| 26 | $M_1$Dor | 1·43 ± 0·27 (11) |
| 25 | $M_2$GeB | 1·53 ± 0·20 (21) |

Significance of excess over $M_1$Dor and $M_2$GeB by *t* test + +$P < 0{\cdot}001$; +$P < 0{\cdot}01$.
The numbers of cultures are shown in parentheses. Mean values ± s.e. are given.

FIG. 3.—Lineweaver–Burk plots of DO. Two differents subcultures (x, o) of the same melanoma cell line were incubated in the reaction mixture described in Material and Methods. Concentrations of [1·4-$^{14}$C] putrescine were varied from 1·25 to 50 × $10^{-6}$M. 1/S = $10^{-6}$/[putrescine] 1/V = 1/pmol $\Delta^1$-pyrroline formed/h/0·1 mg protein. A, $M_3$Dau cell line; B, $M_4$Beu cell line.

$10^{-2}$M chloral hydrate. The results in Fig. 4 show that for the highly tumorigenic lines the $K_m$ (app.) values are 10·6 × $10^{-6}$M ± 0·2 (5) and 14·2 × $10^{-6}$M ± 0·6 (5) respectively, whereas for the poorly tumorigenic lines they are 4·5 × $10^{-6}$M ± 0·3 (8). Thus DO from highly tumorigenic lines has a greater apparent $K_m$ than that from the poorly tumorigenic lines. These findings prompted us to examine the kinetic parameters of DO from normal and transformed rat kidney

FIG. 4.—Lineweaver–Burk plots of DO in different melanoma cell lines. Concentrations of [1·4-$^{14}$C] putrescine were varied from 1·25 to 50 × 10$^{-6}$M. 1/S = 10$^{-6}$/[putrescine]; 1/V = 1/pmol $\Delta^1$-pyrroline formed/h 0·1 mg protein. H, $M_2$GeB line; $M_1$Dor; □, $M_4$Beu; ×, $M_3$Dau.

FIG. 5.—Lineweaver–Burk plots of DO from $M_1$Dor melanoma cells (——) and cells established from tumours appearing in nude mice after injection of $M_1$Dor cells (—·—·). Concentration of [1·4-$^{14}$C] putrescine was varied from 2 to 20 × 10$^{-6}$M. 1/S = 10$^{-6}$/[putrescine]. 1/V = 1/pmol $\Delta^1$-pyrroline formed/h/0·1 mg protein.

cells, in which residual DO activity at confluence was lower in the cells transformed by Rous sarcoma virus than in their normal counterparts. It was found here too that the $K_m$ (app.) of DO from the transformed rat kidney cells is $2{\cdot}5 \times 10^{-6}$M, compared to $5{\cdot}5 \times 10^{-7}$M in the normal cells. It thus seems reasonable to conclude that an increased $K_m$ (app.) is characteristic of both highly tumorigenic and transformed cells.

In view of these results, plus the findings of Kanzaki *et al.* (1979) and Brüggen *et al.* (1981) that a population of melanoma cells is heterogeneous, the question could be asked whether the low $K_m$ (app.) in the poorly tumorigenic lines was characteristic of the enzyme from all the melanoma cells in the population, or whether it represented the overall value of a heterogeneous population containing a mixture of highly tumorigenic and poorly tumorigenic cells. To assess this, cell cultures were established from tumours appearing in nude mice after injection of the poorly tumorigenic line $M_1$Dor.

Determination of $K_m$ (app.) of DO from these tumour cells revealed that it was $14 \times 10^{-6}$M, whereas that of the original poorly tumorigenic line was $4{\cdot}5 \times 10^{-6}$M (Fig. 5). Thus this melanoma line does contain a mixture of highly tumorigenic cells (responsible for tumour formation) and of poorly tumorigenic ones. The results provide further evidence for the correlation established so far between DO and tumorigenicity. It must be noted that the $K_m$ (app.) of DO shows no variation in $M_3$Dau (which exhibits 100% tumorigenicity in nude mice) whether the cultures were established from human or mouse tumours. This suggests that the cell population in the poorly tumorigenic line is heterogeneous, and that the more tumorigenic cells of this population have a $K_m$ (app.) typical of the 100% tumorigenic lines.

### DISCUSSION

It is apparent from the results presented that modifications in DO activity take place with the growth of human malignant melanoma cells in culture, as has been shown for rat kidney cells. Contrary to observations made in studies comparing DO activity in homogenates of confluent cultures from normal rat kidney cells and from these same cells transformed by the wild-type strain of Rous sarcoma virus B77, the DO activity, expressed as pmol $\Delta^1$-pyrroline formed/h/0·1 mg protein, in confluent cells from the highly malignant lines is greater than that of the less malignant lines. Though these results were statistically significant (Table) they must be interpreted with caution, since $V_{max}$ (app.) values obtained on kinetic analysis showed that this parameter varied with the number of subcultures undergone by the cell line (Fig. 3) and also with the presence of dialysable modulators of the enzyme (unpublished results). Evidence for such natural modulators of DO has been previously presented (Quash *et al.*, 1976) and whereas 2-oxosuccinamate derived from asparagine transamination was an activator, the deaminated derivative of this ketoacid oxaloacetate, and its decarboxylated product pyruvate, were inhibitors. Variations in the intracellular level of such modulators with passage number and culture conditions could provide one explanation for the differences in $V_{max}$ described here. They could also explain the apparently conflicting findings, showing on the one hand increased DO activity in human malignant cells from lung (Baylin *et al.*, 1980) and thyroid (Andersson *et al.*, 1980), and on the other decreased DO activity in malignant cells from oesophagus (Quash *et al.*, 1979) and intestine (Kusche *et al.*, 1980).

The investigation of the other kinetic parameter, $K_m$ (app.), gave results which did not fluctuate either with the number of subcultures (Fig. 3) or with the dialysis or not of cell homogenates. Using this criterion, the 4 melanoma cell lines were classified into 2 groups of high and low $K_m$ (app.) and this classification corresponded to the highly tumorigenic and low tumorigenic lines respectively. Using this same parameter, it was found that the $K_m$ (app.) of DO from rat kidney cells transformed by Rous sarcoma virus showed a 20-fold increase over that of the enzyme from normal rat kidney cells. Thus even if increases or decreases in enzyme activity and $V_{max}$ (app.) take place in tumour cells according to their growth phase and/or anatomical origin, it would appear that an increased $K_m$ (app.) of DO is characteristic of all tumour cells.

This example of an altered $K_m$ of an enzyme in tumour cells is not an isolated observation. White *et al.* (1981) have very recently provided evidence for an altered $K_m$ in the sugar-transport system for deoxyglucose in tumour cells. Moreover the character of an altered $K_m$ for hexose transport segregates as a character of tumorigenicity in hybrids made between malignant and normal cells. In fact using heterotransplantability in syngeneic hosts as the assay for tumorigenicity, they were further able to show a definite correlation between tumorigenicity and an altered $K_m$ in the enzyme system responsible for deoxyglucose transport. Other examples of enzymes with altered kinetic properties in human neoplastic tissues have been documented for 7 melanoma cultures xenografted into athymic nude mice. In such cultures Liau *et al.* (1980) demonstrated the presence of S-adenosyl-methionine synthetase isoenzyme with an altered intermediate $K_m$ which was attributable to the malignant evolution of the cells. Such an alteration is analogous to that found for the leucine aminotransferase isoenzymes reported by Roth & Kaji (1979). The transition of normal isoenzyme I into tumour isoenzyme III for leucine aminotransferase is caused by the transforming-gene product of Rous sarcoma virus. The alteration of isoenzymes in malignant tissues appears to be a general phenomenon.

In the results of all the studies cited above, as well as in those presented here, there was one inherent difficulty: the validity of the correlation between enzyme kinetics and tumorigenicity depended on the homogeneity of the cell population used. A cell population may be heterogeneous and be composed of cells with different tumorigenic potential in nude mice. With cell lines exhibiting 100% take or no take at all in nude mice the cell population is most probably homogeneous, and the interpretation is straightforward. For those showing intermediate percentages, differences may reflect the heterogeneity of the cell population rather than an altered characteristic of the enzyme from each cell. This possibility was investigated directly by examining the $K_m$ (app.) of DO in cells of the poorly tumorigenic line $M_1$Dor before and after transplantation into nude mice. Since cells growing in these animals are by definition tumorigenic, a population of tumorigenic cells can then be selected from a poorly tumorigenic cell line. The fact that tumours arising under these conditions result from population selection in nude mice was documented by their karyotype, which contained multiples of chromosomes 3, 7, 16 and 22, relatively few copies of chromosomes 9 and 21, and was similar to that of highly tumorigenic melanoma cell lines (Bertrand *et al.*, in preparation). When kinetic studies were performed on DO in cells subcultured from xenografts, it was found that the $K_m$ (app.) of DO was typical of a highly tumorigenic line ($K_m$ (app.) $14 \times 10^{-6}$M), whereas the $K_m$ of the enzyme in the cell population from which the cells were selected was $4{\cdot}5 \times 10^{-6}$M. This result provides evidence not only for the heterogeneity of the cell population, but gives additional support to the observations made with $M_3$Dau and $M_4$Beu, that increased tumorigenesis is accompanied by an increase in the $K_m$ of DO.

The basis of this altered $K_m$ of DO will not be fully understood until the enzyme is rigorously purified and appropriate antibodies have been made. Nevertheless the pursuit of investigations along the lines outlined here with other xenografted human tumours should allow conclusions to be reached as to whether increases in $K_m$ (app.) of DO are a general characteristic of all tumorigenic cells.

We would like to thank Mrs H. Ripoll for her excellent technical assistance, Mrs H. Cabrillat for help with the heterotransplantation assays and Mrs S. Bertrand for karyotype studies. We are indebted to Dr L. Gazzolo for stimulating discussion and to Dr J. Huppert for his interest in this work.

This research was supported by a grant from the Délégation Générale à la Recherche Scientifique et Technique No 78-7-2650 and partly by Grants from F.é.G.E.F.L.U.C. and Fédération des Centres de Lutte Contre le Cancer.

### REFERENCES

ANDERSSON, A. C., HENNINGSSON, S. & ROSENGREN, E. (1979) The influence of some drugs on the determination of diamine oxidase activity. *Agents Act.* **9**, 44.

ANDERSSON, A. C., HENNINGSSON, S. & JÄRHULT, J. (1980) Diamine oxidase activity and γ-aminobutyric acid formation in medullary carcinoma of the thyroid. *Agents Act.* **10**, 299.

BAYLIN, S. B., BEAVEN, M. A., BUTA, L. M. & KEISER, H. R. (1972) Histaminase activity: A biochemical marker for medullary carcinoma of the thyroid. *Am. J. Med.*, **53**, 723.

BAYLIN, S. B., ABELOFF, M. D., GOODWIN, C., CARNEY, D. N. & GAZDAR, A. F. (1980) Activities of L-Dopa decarboxylase and diamine oxidase (histaminase) in human lung cancers and decarboxylase as a marker for small (oat) cell cancer in cell culture. *Cancer Res.*, **40**, 1990.

BRÜGGEN, J., MACHER, E. & SORG, C. (1981) Expression of surface antigens and its relation to parameters of malignancy in human malignant melanoma. *Cancer Immunol. Immunother.*, **10**, 121.

DON, S. & BACHRACH, U. (1975) Polyamine metabolism in normal and virus-transformed chick embryo fibroblasts. *Cancer Res.*, **35**, 3618.

GIOVANELLA, B. C., STEHLIN, J. S., SANTAMARIA, C. & 6 others (1976) Human neoplastic and normal cells in tissue culture. I. Cell lines derived from malignant melanomas and normal melanocytes. *J. Natl. Cancer Inst.*, **56**, 1131.

JACUBOVICH, R. & DORÉ, J. F. (1979) Tumour associated antigens in culture medium of malignant melanoma cell strains. *Cancer Immunol. Immunother.*, **7**, 59.

JÄNNE, J., PÖSÖ, H. & RAINA, A. (1978) Polyamines in rapid growth and cancer. *Biochim. Biophys. Acta*, **473**, 241.

KALLIO, A., PÖSÖ, H., GUHA, S. K. & JÄNNE, J. (1977) Polyamines and their biosynthetic enzymes in Ehrlich ascites-carcinoma cells. *Biochem. J.*, **166**, 89.

KANZAKI, T., HASHIMOTO, K. & BATH, D. W. (1979) Heterotransplantation of human malignant melanoma cell lines in athymic nude mice. *J. Natl Cancer Inst.*, **62**, 1151.

KOBAYASHI, Y. (1963) Blood levels of diamine oxidase as a test for pregnancy. *J. Lab. Clin. Med.*, **62**, 699.

KUSCHE, J., BIEGANSKI, T., HESTERBERG, R. & 4 others (1980) The influence of carcinoma growth on diamine oxidase activity in human gastrointestinal tract. *Agents Act.* **10**, 110.

LIAU, M. C., CHANG, C. F. & GIOVANELLA, B. C. (1980) Demonstration of an altered S-adenosylmethionine synthetase in human malignant tumors xenografted into athymic nude mice. *J. Natl Cancer Inst.*, **64**, 1071.

LIN, C. U., ORCUTT, M. L. & STOLBACH, L. L. (1975) Elevation of histaminase and its concurrence with Regan isoenzyme in ovarian cancer. *Cancer Res.*, **35**, 2762.

O'BRIEN, T. G. (1976) The induction of ornithine decarboxylase as an early possibly obligatory event in mouse skin carcinogenesis. *Cancer Res.*, **36**, 2644.

QUASH, G. A., CALOGERO, H., FOSSAR, N., FERDINAND, A. & TAYLOR, D. (1976) Modification of diamine oxidase activity *in vitro* by metabolites of asparagine and differences in asparagine decarboxylation in normal and virus-transformed baby hamster kidney cells. *Biochem. J.*, **157**, 599.

QUASH, G. A., KEOLOUANGKHOT, T., GAZZOLO, L., RIPOLL, H. & SAEZ, S. (1979) Diamine oxidase and polyamine oxidase in normal and transformed cells. *Biochem. J.*, **177**, 275.

ROTH, S. L. & KAJI, A. (1979) Change of isoenzyme protein during activation of a transforming gene product: Its relation to other biochemical markers of cellular transformation and differentiation. *Biochem. Biophys. Acta*, **583**, 241.

RUSSELL, D. H. & SNYDER, S. H. (1968) Amine synthesis in rapidly growing tissues: Ornithine decarboxylase activity in regenerating rat liver, chick embryo and various tumors. *Proc. Natl Acad. Sci.*, **60**, 1420.

SCALABRINO, G., PÖSÖ, H., HANNONEN, P., KALLIO, A. & JÄNNE, J. (1978) Synthesis and accumulation of polyamines in rat liver during chemical carcinogenesis. *Int. J. Cancer*, **21**, 239.

SESSA, A., DESIDERIO, M. A., BAIZINI, M. & PERIN, A. (1981) Diamine oxidase activity in regenerating rat liver and in 4-di-methylaminoazobenzene-induced and Yoshida AH130 hepatomas. *Cancer Res.*, **41**, 1929.

STILES, C. D., DESMOND, W., CHUMAN, L. M., SATO, G. & SAIER, M. H. (1976) Relationship of cell growth behaviour *in vitro* to tumorigenicity in athymic nude mice. *Cancer Res.*, **36**, 3300.

WHITE, M. K., BRAMWELL, M. E. & HARRIS, M. (1981) Hexose transport in hybrids between malignant and normal cells. *Nature*, **294**, 232.